# Anxiety-like behaviour increases safety from fish predation in an amphipod crustacea

**DOI:** 10.1098/rsos.171558

**Published:** 2017-12-06

**Authors:** Marie-Jeanne Perrot-Minnot, Loan Banchetry, Frank Cézilly

**Affiliations:** Laboratoire Ecologie Evolution, UMR6282 CNRS, Université Bourgogne Franche-Comté, Dijon 21000, France

**Keywords:** anxiety, defensive behaviour, glutamate, neuromodulation, predation

## Abstract

Anxiety is an emotional state generally expressed as sustained apprehension of the environment and elevated vigilance. It has been widely reported in vertebrates and, more recently, in a few invertebrate species. However, its fitness value remains elusive. We investigated anxiety-like behaviour and its consequences in an amphipod crustacean, using electric shock as aversive stimuli, and pharmacological assays. An anxiety-like state induced by electric shocks in *Gammarus fossarum* was expressed through increased sheltering behaviour in the absence of predation risk, thereby showing the pervasive nature of such behavioural response. Increasing the number of electric shocks both increased refuge use and delayed behavioural recovery. The behavioural effect of electric shock was mitigated by pre-treatment with LY354740, a metabotropic glutamate receptor group II/III agonist. Importantly, we found that this modulation of decision-making under an anxiety-like state resulted in an increased survival to predation in microcosm experiments. This study confirms the interest in taking an evolutionary view to the study of anxiety and calls for further investigation on the costs counterbalancing the survival benefit of an elevated anxiety level evidenced here.

## 1. Introduction

Most living organisms regularly experience acute stress, particularly when facing unpredictable or transient threats in their environment, such as a sudden change in temperature, toxic food or the presence of a predator. Response to stress is considered to be adaptive when it decreases the risk of death or injury and facilitates coping mechanisms against future stressors [1]. In particular, it has been shown that various types of stressors can generate fear and anxiety in animals. Fear is generally regarded as a basic response to a real or well-identified threat that generates an immediate response such as fleeing, freezing or aggression. By contrast, anxiety is generally regarded as a more complex phenomenon that results from uncertainty about potential future threats [2]. Under this emotional state, individual perception (assessment) of threat cost and probability are inflated [2], thereby inducing a sustained apprehension of the environment and elevated vigilance [3]. Anxiety differs from fear because it persists in the absence of the stressor and in a new context [4,5], and because of the uncertainty regarding the likelihood, timing or nature of a future threat [2]. The experimental study of anxiety has initially been limited to human beings, primates [6] and a few other vertebrates, especially rodents [7]. However, recent advances have shown that the concept probably applies to all vertebrates [8] and possibly extends to invertebrates [6,9–12]. For instance, anxiety-like behaviour has been evidenced in the crayfish (*Procambarus clarkii*) following exposure to stress [5] or to social harassment [13]. Although the adaptive value of fear in animals is largely acknowledged [14,15] and, more recently, of nociceptive sensitization [16], evidence for adaptive or maladaptive anxiety remains scarce (see, however, [17]).

Here, we extend the study of anxiety-like behaviour to another crustacean species with a twofold objective: (i) to assess how common this feature is among invertebrates, and (ii) to estimate the fitness value of anxiety. We first investigated whether the freshwater amphipod *Gammarus fossarum* expresses anxiety-like behaviour, through combining behavioural and physiological assays. To stress the animals, we subjected gammarids to electric shocks [5,18,19]. We then assessed whether the stress-induced behavioural response is context independent, increases with the level of stress experienced and persists in the absence of stressor, as expected in the case of induced anxiety [2,5]. The behavioural response recorded to quantify the anxiety level was the use of a refuge, a typical antipredator behaviour of crustacean amphipods, known to be influenced by olfactory cues from fish predators [20–22]. We next assessed whether the effect of electric shock on refuge use was mitigated by prior anxiolytic treatment. Previous studies on anxiolytic and antidepressant effects of neuro-pharmacological drugs on crustaceans have mainly targeted the serotonergic system [11,13,23,24] or the GABAergique system [5]. Here, we choose to test the efficacy of a new-generation anxiolytic, that is, an agonist of metabotropic glutamate receptor (mGluR) [25,26]. LY354740 is an agonist of group II and III metabotropic glutamate receptors (mGluR II/III), with a potent and selective anxiolytic effect in vertebrates [27]. However, so far, its anxiolytic effect in invertebrates has not been documented.

As strong evidence for a stress-related anxious state in *G. fossarum* was obtained, we further estimated its fitness consequences. To that aim, we conducted predation experiments in microcosms, comparing vulnerability to predation between anxious and control individuals. We quantified the vulnerability to predation as the number of prey captured by a fish predator in a limited amount of time, out of a group of gammarid prey previously exposed to an electric shock or not (controls). In predation experiments with amphipods, several confounding variables must be controlled for. First, gammarids respond to olfactory cues released from predators [22,28–31]. The immediate perception of such cues might constitute a confounding source of stress during the predation test, in both experimental and control groups. Therefore, we controlled for predator-released olfactory cues, by using a fish predator towards which gammarids were naive, the goldfish, *Carassius auratus* [32]. Second, the social context under which individuals are exposed to predation risk may modulate their behavioural response. For instance, it has been shown that conspecific aggregation increases under exposure to olfactory cues from predators [30,31]. Sheltering behaviour in response to the level of risk may, therefore, interact with the social context under which the individual is tested, alone or in a group. Therefore, we assessed the effect of anxiety on refuge use in groups prior to predation tests, in a separate experiment using the same experimental arena. Gammarids in the group showed a similar anxiety-like state to that of individual ones. Importantly, groups of *G. fossarum* treated with electric shock were less vulnerable to predation by goldfish compared to control groups. Taken together, our results show that an anxiety-like state can be induced in this invertebrate species and reveal the fitness benefit of this anticipatory emotional state in a predation context. The possible costs counterbalancing the survival benefit of elevated anxiety level are discussed.

## 2. Material and methods

### 2.1. Animals

*Gammarus fossarum* individuals were collected in the Norges River, Burgundy, France (47°21′40.2′′ N 5°09′30.6′′ E), from March 2015 to June 2016. To control for sex-related differences in the response to predation risk [29], only males were kept for experiments. They were left in acclimatization for at least one week before the experiments started, in tanks filled with self-renewed oxygenated dechlorinated ultraviolet (UV)-treated water (conditioned water (CW)), and placed in a temperature- and photoperiod-controlled room (16°C, UV, 12 L : 12 D). Gammarids were provided with decayed leaves as food and river substrate.

Goldfish used in the predation experiments were between 8 and 12 cm in length. They were purchased from a pet shop and maintained in the laboratory in large tanks (100 l) filled with oxygenated dechlorinated UV-treated water. During acclimatization, goldfish were trained to chase and prey on gammarids by providing only live gammarids as food. To control for the release of olfactory cues potentially signalling predation risk during experiments, goldfish were kept fasting for 48 h prior to predation tests. Fish were individually identified by one or several distinctive phenotypic traits (colour spot pattern) and used once per type of prey in a randomized order.

### 2.2. Experimental modulation of anxiety in gammarids

For chronic anxiolytic treatment, we used the metabotropic glutamate receptor group II/III agonist LY354740 (L1045, Sigma Aldrich, St Louis, MO, USA; thereafter LY). LY has a limiting function on glutamate release in glutamatergic synapses [27]. It is expected to have potent and selective anxiolytic effect, with fewer side effects compared to benzodiazepines drugs acting on the GABA_A_ receptor [27], and is considered as a promising drug in the treatment of anxiety- and stress-related disorders in humans [33]. Gammarids were maintained in the LY solution for two weeks in groups of 10 in plastic containers (150 ml), provided with decaying leaves and a few stones as refuge. Solutions at two different concentrations of LY were used in two separate experiments: 10 µg l^−1^ and 100 µg l^−1^, and changed every 48 h. Control individuals were maintained in the same way, but were not exposed to LY. Anxiolytic treatment was delivered before exposure to electric shock.

**Figure 1.**
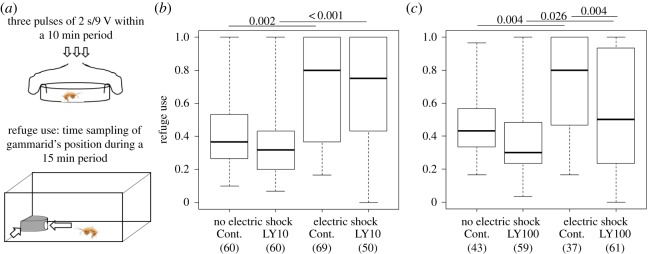
Anxiety-like behaviour in gammarids: effect of exposure to electric shock and prior anxiolytic treatment on refuge use of individual gammarids. (*a*) Experimental design to expose gammarids to electric shocks, and to record refuge use. (*b,c*) Effect of electric shock and chronic exposure to the anxiolytic LY354740 at 10 µg l^−1^ (LY10) (*b*), and at 100 µg l^−1^ (LY100) (*c*). Cont., controls for chronic exposure to the anxiolytic. Box plots represent the median, first and third quartiles and min–max. Sample sizes are provided below group labels. *p*-values following post hoc rank test for multiple comparisons (Dunn's test) are given above bars, unless non-significant (see the electronic supplementary material, table S1 for statistical details).

To induce an anxiety-like state, we exposed gammarids to electric shocks prior to behavioural testing as electric shocks have been proven to be noxious or even painful in crustaceans [5,18,19,28]. An electric system was designed to deliver short impulses of 50 Hz (9 V) in a Petri dish (figure 1*a*). Gammarids were individually exposed to three electric pulses of 2 s delivered at 5 min intervals for 10 min. Control gammarids were also placed in the same conditions but did not receive the electric stimulation. We observed that gammarids reacted to electric shocks by performing kicking movements immediately following electric pulses, a behaviour similar to escape response when evading the attack of a conspecific or that of a predator. This movement seems quite analogous to the escape tail flicks reported in crayfish [5]. In agreement with other studies showing avoidance and aversive learning to shock in crustaceans [19,34,35], such observations strongly suggest that electric shocks were perceived as a noxious stimulus in gammarids.

### 2.3. Behavioural experiments

#### 2.3.1. Individual refuge use and locomotor activity

The sheltering behaviour of individual gammarids was recorded by quantifying refuge use according to Perrot-Minnot *et al.* [36], with modifications. Individual gammarids were placed in a box filled with 300 ml CW, offering an opaque refuge (consisting of half a terracotta saucer of 8.5 cm on its larger side, with a 1 cm^2^ opening) covering about 18% of the surface on one side, exposed to a light of 1.3 Klux (anxiolytic experiments) or 0.9 Klux (dose and remanence experiment, see below). After an acclimatization time of 5 min, the position of the individual gammarid was recorded by scan sampling every 30 s for 15 min, and scored as 1 (outside) or 0 (under the refuge). Refuge use was quantified as the number of times individual gammarids were under refuge out of the maximum score (30) and, therefore, reported as a frequency.

Locomotor activity was recorded just after scoring refuge use, following [36]. Real-time automated recording of swimming activity was performed in a Petri dish filled with CW without substrate, under moderate light intensity (400 lux). After an acclimatization time of 3 min, time spent below and above a speed threshold of 15 mm s^−1^ was recorded for 3 min using the Lighting infrared system and an infrared camera connected to a laptop (Zebralab software, View Point^®^, Lyon, France). Preliminary tests showed that below 15 mm s^−1^, gammarids locomotion is achieved by crawling rather than swimming.

#### 2.3.2. Dose effect and remanence of exposure to electric shocks

To further characterize anxiety-like response in gammarids, we addressed the potential dose-dependent effect of stress on anxiety level, the persistence of an anxiety-like state and the relationship between persistence and the initial level of stress. We recorded the use of refuge in gammarids exposed to a different number of electric shocks within a 10 min period, from 5 to 180 min after exposure to stress. Stress exposure was performed as described above, except that the number of electric shocks delivered in a 10 min period was 1, 3 or 6. Refuge was recorded as above, 5, 45, 90 and 180 min, after stress. The position of the refuge in the box was switched between two consecutive records (and its initial position alternated from one series to another) to avoid familiarization. This procedure was necessary to evaluate the persistence of stress-induced behavioural changes, as a criterion for anxiety and a necessary condition to run 25 min long predation tests (see below).

#### 2.3.3. Individual refuge use in response to a familiar predatory fish

To compare the effect size of exposure to electric shock to an ecologically relevant stress, we analysed data from a previous experiment in which refuge use by *G. fossarum* from the same population, in response to olfactory cues from brown trout, *Salmo trutta*, was recorded. The same experimental design was used, except that refuge use was scored every 3 min for 1 h. Refuge use was scored in the absence of olfactory cues from trout (controls), or in water from the tank where trout were maintained at a biomass ratio of 10 g l^−1^ and fed on live gammarids.

#### 2.3.4. Group refuge use

To assess refuge use in groups of gammarids, 10 gammarids, either stressed or unstressed, were placed together in a tank, using the same experimental arena as the one used for predation trials. The opaque refuge was made of a half-cup of clay (same as the one used for individual refuge use, see above) placed upside down in the middle of a plastic tank (13 × 22 × 12.5 cm), thereby offering a large opening on one side (8.5 cm length, 1.2 cm height) and a small opening on the other (1 cm^2^). The tank was filled with 1.5 l of UV-treated water and surrounded by an opaque black plastic bag to avoid any disturbance from the observer. Tanks were exposed to a light of 1.3 Klux. Refuge use in the group was evaluated after a 10 min acclimatization time by recording the number of gammarids out of the refuge at the end of acclimatization time, 15 min later. Duration of the trial was adjusted as to fit the duration of the predation test, to give an estimate of the number of prey accessible for goldfish.

### 2.4. Predation experiment

To evaluate whether increased refuge use effectively reduces susceptibility to predation, groups of 10 gammarids were exposed to a goldfish using the same experimental arena as the one used for refuge use in groups. Groups of 10 gammarids of the same status (i.e. either shocked by exposure to electric impulse or unstressed) were placed in tanks and left for acclimatization for 10 min. One goldfish was then introduced in each tank and left for 15 min. The number of gammarids remaining at the end of the predation test was counted to determine the number of prey eaten by each goldfish. Individual goldfish were identified based on their unique pigmentation pattern, and could therefore be presented in random order to each prey status (control and shocked). Predation tests using the same individual goldfish were run at 2-day intervals (without food) to increase and standardize goldfish motivation to feed.

### 2.5. Statistical analyses

All statistical analyses were performed using the software R (v. 3.2.4) [37]. The distribution of behavioural scores did not conform to normality, nor did the number of prey eaten. We, therefore, relied on non-parametric tests. The effects of LY treatment and stress induced by electric shock on individual refuge use were tested using a Kruskal–Wallis, followed by post hoc paired comparisons accounting for tied ranks (Dunn's test using the Benjamini–Yekuteili method to adjust *p*-values for multiple comparisons, package ‘Dunn.test’ [38]). Locomotor activity was quantified as the proportion of time spent swimming (movement speed above 15 mm s^−1^), and arcsine-transformed to fit normal distribution. The effects of LY treatment and stress induced by electric shock on locomotor activity were then tested using a two-way ANOVA. To test for a tendency to increase refuge use with increasing stress level (number of electric shocks), we used the Jonckheere–Terpstra trend test for ordered alternatives (package ‘DescTools’: [39], alternative hypothesis: increasing, 5000 permutations). The number of prey eaten by goldfish was analysed using Wilcoxon's paired signed-rank tests (controlling for individual fish; package ‘MASS’ [40]). All tests were two-sided and used a significance threshold of *p* < 0.05, unless specified.

Finally, we relied on Cliff's delta as an estimate of the effect size of electric shock on refuge use, activity and vulnerability to predation [36]. The median and 95% CI of Cliff's delta were calculated using the R-package ‘orddom’ [41], with 10 000 bootstraps. Based on the threshold values for Cliff's delta reported in [42], the magnitude of effect sizes was interpreted as negligible (less than 0.147), small (between 0.147 and 0.33), medium (between 0.33 and 0.474) or strong (more than 0.474).

## 3. Results

### 3.1. Anxiety-like behaviour in *Gammarus fossarum*

Gammarids exposed to electric shock significantly increased their use of refuge (two replicated experiments: *p *= 0.002, *p = *0.004, figure 1*b*,*c*; electronic supplementary material, table S1). Prior treatment with the anxiolytic mitigated this behavioural response. Gammarids treated with LY at 100 µg l^−1^ prior to exposure to an electric shock used the refuge to a lower extent than untreated ones (*p = *0.004), albeit still significantly more compared to LY-treated ones unexposed to electric shock (*p *= 0.026) (figure 1*c*; electronic supplementary material, table S1 and figure S1). Their sheltering behaviour after stress exposure was comparable to untreated unexposed gammarids (figure 1*c*; electronic supplementary material, table S1). By contrast, gammarids pre-treated with LY at 10 µg l^−1^ increased their use of refuge in response to exposure to an electric shock at a comparable level as untreated individuals (*p *= 0.77, figure 1*b*; electronic supplementary material, table S1 and figure S1). Prior treatment with LY did not affect the use of refuge by unexposed gammarids, as expected for an anxiolytic treatment in the absence of stress (figure 1*c*; electronic supplementary material, table S1).

To further ascertain that electric shock exposure mainly induces sustained motivational change with negligible physical impairment, we quantified the locomotor activity of gammarids exposed to stress and anxiolytic just after recording refuge use, using a real-time automated recording device. Gammarids spent on average 68% of total time swimming above the speed threshold of 15 mm s^−1^ (±17.7 s.d.). Locomotor activity was not significantly affected by stress nor by anxiolytic treatment in both experiments (LY at 10 µg l^−1^, *F*_3,225_ = 0.89, *p* = 0.45, and LY at 100 µg l^−1^, *F*_3,198_, *p* = 0.95; electronic supplementary material, figure S2).

### 3.2. Anxiety level and recovery time according to stress intensity

**Figure 2.**
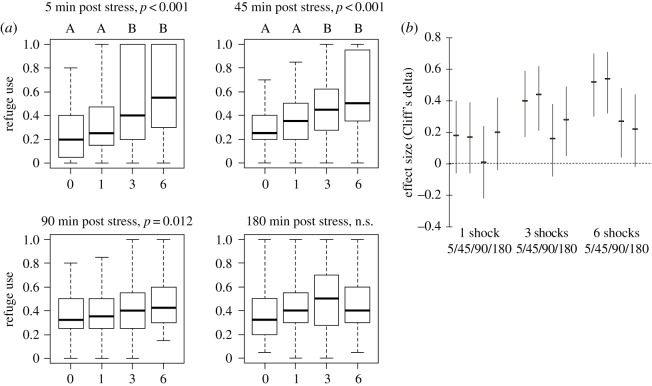
Dose and persistence effect of electric shock on anxiety-like behaviour. Individual gammarids were exposed to one of four levels of stress: 0, 1, 3 or 6 electric pulses of 2 s at 9 V, in a 10 min time period. Refuge use was recorded at 5, 45, 90 and 180 min post-stress as above. Sample size was 48 individuals per level of electric shock. (*a*) Refuge use gradually increase with increasing stress level at 5, 45 and 90 min following stress exposure (the Jonckheere–Terpstra trend test for ordered alternatives, *p*-values above graph). Treatments with the same letter are not significantly different, after post hoc multiple comparison Dunn's test (see the electronic supplementary material, table S2 for statistical details). (*b*) Effect size of exposure to electric shock in stressed gammarids compared to unstressed ones, calculated 5, 45, 90 and 180 min after exposure to stress (median and bootstrapped IC (95%) of Cliff's delta index). Effect sizes of exposure to three or six shocks are medium to strong, respectively, until at least 45 min post-stress, and are small and marginally significant at 180 min and 90 min, respectively, suggesting that recovery from stress occur between 45 and 90 min post-stress.

Enhancing the level of stress by increasing the number of electric shocks delivered in a 10 min period from 1 to 6 resulted in a gradual increase in refuge use (figure 2*a*). This trend was evidenced at 5, 45 and 90 min, following stress exposure (the Jonckheere–Terpstra trend test for ordered alternatives, JT = 9056, *p *< 0.001; JT = 9079, *p *< 0.001 and JT = 7870.5, *p *= 0.012, respectively), but was not significant at 180 min post-stress (JT = 7603, *p *= 0.052; figure 2*a*). Exposure to stress induced a significant change in refuge use at 5 and 45 min post-stress (Kruskall–Wallis: *z* = 26.27, *p* < 0.0001 and *z* = 26.98, *p* < 0.0001, respectively), but not at 90 min nor at 180 min post-stress (*z* = 7.32, *p* = 0.06 and *z* = 6.66, *p* < 0.08, respectively). Gammarids exposed to three or six electric shocks displayed a comparable and significant increase in refuge use at 5 and 45 min after exposure, compared to unstressed ones (*p* = 0.001 and *p* < 0.0001, respectively), whereas a single shock failed to elicit a behavioural response (figure 2*a*; electronic supplementary material, table S2). Recovery from stress was nearly complete from 90 min post-stress onwards (figure 2*a*), although effect sizes of exposure to three and six shocks were still non-null at 180 and 90 min, respectively (figure 2*b*).

### 3.3. Anxiety-like state and vulnerability to predation

**Figure 3.**
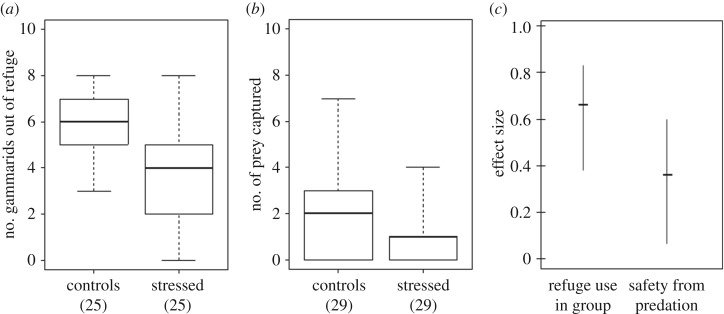
Consequence of anxiety-like behaviour on the vulnerability to predation. (*a*) Anxiety-like behaviour in groups of gammarids exposed to electric shock (stressed) is expressed through an increased number of gammarids seeking a refuge compared to unstressed control ones (25 min post-stress); (*b*) number of prey captured by goldfish within a 15 min time period, out of groups of 10 stressed or 10 unstressed (controls) gammarids. (*a,b*) Box plots represent the median, first and third quartiles and min–max. Sample sizes are provided below group labels. (*c*) Effect size of stress induced by electric shock on refuge use in group, and on the number of prey captured by goldfish (median and bootstrapped IC (95%) of Cliff's delta index): positive value corresponds here to an increase in the use of refuge, and an increased safety from predation, compared to unstressed individuals.

In agreement with anxiety-like changes in refuge use of isolated gammarids, stressed gammarids in groups used the refuge more than unstressed ones, both at the beginning of the test (*W* = 511.5, *p* < 0.0001; figure 3*a*) and at the end of the trial 15 min later (*W* = 489, *p* = 0.0006). In predation trials, the number of prey captured was significantly lower when goldfish were provided with stressed gammarids than with control ones (*V* = 196.5, *p* = 0.005; figure 3*b*). The effect size of stress induced by electric shock on the number of prey captured was moderate (0.36 ± 0.14 s.e.), and lower than that of stress on group refuge use (0.66 ± 0.11 s.e), albeit not significantly (*z* = 1.68, *p *= 0.20; figure 3*c*).

## 4. Discussion

We designed behavioural and physiological assays in a crustacean amphipod to modulate anxiety-like defensive behaviour, and assessed its survival value in microcosm predation experiments. The modulation of anxiety by stressful experience and anxiolytic treatment translated into variable levels of refuge use in the absence of predation risk. The use of refuge responded in proportion to the level of stress experienced, with enhanced and prolonged sheltering behaviour in response to increasing number of electric shocks. The mGluR II/III agonist LY354740 at 100 µg l^−1^ mitigated this defensive response to electric shock, as the sheltering behaviour of gammarids after stress exposure was comparable to that of untreated, unexposed gammarids. Interestingly, there was no concomitant change in locomotor activity after exposure to electric shock or anxiolytic treatment. This suggests that the electric shocks delivered here did not impair muscle function, and that the mGluR II/III agonist anxiolytic did not induce the sedative effect reported for anxiolytic targeting GABA_A_ receptors (benzodiazepines) [44]. Taken together, we interpret the change in sheltering behaviour as an emotional expression of anxiety by *G. fossarum*, rather than the impairment of movement.

Behavioural responses follow decision-making rules, driven by the interaction between the internal state of the organism and environmental cues or challenges it may perceive. Predation risk is a pervasive source of stress in the daily life of organisms [44,45], such that the benefit accruing from anxiety might be directly related to reduced vulnerability to predation. Here, an anxiety-like state reduced predation by an unfamiliar fish predator, thereby revealing a potential fitness benefit of this emotional state. Interestingly, our results are comparable to the increased survival to predation reported in squid following long-term nociceptive sensitization (although the term anxiety was not used by the authors) [16]. From an ecological view, the inducible nature of anxiety and its moderate sustainability suggest that the benefits from anxiety-induced behavioural response might be counterbalanced by costs [46]. The downside of increased anxiety could include pathological side effects, including cognition impairments, as evidenced in rodents and humans [47]. Additionally, ecological costs associated with anxiety-like state may accrue from a reduced ability to find food or mates [44], as a consequence of decreased foraging behaviour, increased neophobia or reduced sociability. Gammarid amphipods are a particularly suited model to address the existence of such counterbalancing costs, as they show context-dependent mating behaviour, food-intake rate and aggregation to conspecifics, in response to predation risk [21,30,48].

The pervasive nature of anxiety across the animal kingdom also raises the question of its underlying neurophysiological regulation. Interestingly, striking similarities between invertebrates (crayfish) and vertebrates in the chemical pathways linked to anxiety have been recently evidenced. Several studies support a pivotal role for serotonergic modulation of anxiety-related behaviour both in vertebrates [49] and in invertebrates [5,9,11,50]. Physiological manipulations have revealed a complex role for serotonin, with both anxiogenic and anxiolytic effects, possibly related to acute versus chronic exposure to serotonergic drugs depending upon the behaviour under study [9,49]. Another neurotransmitter regulating anxiety is γ-aminobutyric acid (GABA), targeted by the most widely used anxiolytic drugs [5,51]. More recently, the involvement of metabotropic glutamate receptors in regulating anxiety has been evidenced in vertebrates [52]. Here, the efficiency of the glutamate metabotrophic agonist LY354740 as an anxiolytic in the amphipod *G. fossarum* further documents the multisystemic neuromodulation of anxiety, and highlights the glutamatergic system as another pathway, conserved from invertebrates to vertebrates. Interestingly, group II and III mGluR modulate the pre-synaptic release of both glutamate and GABA, and might thereby regulate anxiety by altering the inhibitory/excitatory equilibrium between GABA and glutamate, respectively. Further studies should therefore take a multisystemic view of brain neurotransmitter homeostasis, to address whether unbalanced levels of glutamate/GABA are responsible for anxiety-like behaviour, and how this interacts with the serotonergic system. They should also consider multiple behavioural traits to better assess trait-dependent anxiogenic versus anxiolytic effects [49]. Interestingly, serotonin injection in *G. fossarum* impacts several behavioural traits with varying effect size [36], making this species a particularly valuable model for neuro-ecological studies of anxiety.

In conclusion, our study provides experimental evidence for the survival value of anxiety-like motivational state and broadens the range of species in which anxiety has been evidenced to an amphipod crustacean. To our knowledge, only one previous study provided evidence for similar adaptive benefits [16]. Interestingly, the same fitness benefit, increased survival to predation risk, follows from exposure to different noxious stimuli and in two different taxa (physical injury and mollusc [16], versus electric shock and crustacea in this study). The existence of anxiety-related defensive/protective behaviour in *G. fossarum* suggests that this emotional state could actually be a widespread response induced by stressful experience [4,5,9,11,12], thus opening the way for a comparative framework addressing both the evolutionary significance of anxiety [46] and its underlying mechanisms (see, for instance [12]). Furthermore, our findings on a model species in freshwater ecotoxicology might be relevant for environmental monitoring and the assessment of neuromodulatory disruption in aquatic ecosystems.

## Supplementary Material

Detailed statistical analysis, and effect size of electric shock and anxiolytic treatment on refuge use

## Supplementary Material

Effect of electric-shock and anxiolytic treatments on locomotor activity

## Data accessibility

Data are available from the Dryad Digital Repository: http://dx.doi.org/10.5061/dryad.3kj5s [53].
