# Supplementary material for: Anxiety-like behaviour increases safety from fish predation in an amphipod crustacea
Source: R Soc Open Sci. 2017 Dec 6;4(12):171558. doi: 10.1098/rsos.171558 (PMC5750038; doi:10.1098/rsos.171558)
Supplement: Detailed statistical analysis, and effect size of electric shock and anxiolytic treatment on refuge use [file rsos171558supp1.docx]

*Royal Society Open Science*

Supporting Information

Anxiety-like behaviour increases safety from fish predation in an amphipod crustacea

Marie-Jeanne Perrot-Minnot*, Loan Banchetry, Frank Cezilly

**Appendix 1**: Detailed statistical analysis, and effect size of electric shock and anxiolytic treatment on refuge use

**Table S1**: **Effect of electric shock and anxiolytic treatment on refuge use**. Two separate experiments were run, one with LY at 10 µg.L^-1^ plus control, and one with LY at 100 µg.L^-1^ plus control. The influence of stress induced by electric shock (ES), and of anxiolytic treatment, on refuge use was assessed using the Kruskall-Wallis rank test followed by Dunn post-hoc multiple comparison test (R package ‘Dunn’). The effect of LY354740 concentration was assessed using the Mann-Whitney rank test (package ‘Stats’).

|  | Experiment with LY at 10 µg.L^-1^ | Experiment with LY at 100 µg.L^-1^ |
| --- | --- | --- |
|  | *z* ; *P* | *z* ; *P* |
| Effect of stress (ES) |  |  |
| Individuals unexposed to LY | 3.28 ; **0.002** | 3.28 ; **0.004** |
| LY - exposed individuals | 5.14 ; **< 0.0001** | 2.45 ; **0.026** |
| Effect of prior LY exposure |  |  |
| without stress | 1.73 ; 0.12 | 1.83 ; 0.10 |
| in stressed (ES) individuals | -0.47 ; 0.77 | 3.16 ; **0.004** |
| Effect of LY concentration * |  | |
| without stress | *W* = 1754.5, *P* = 0.94 | |
| in stressed (ES) individuals | *W* = 1927, ***P* = 0.01** | |

**Table S2**: **Dose effect and remanence of electric shock treatment on refuge use**. The effect of the number of electric shocks on refuge use was assessed using the Kruskall-Wallis rank test followed by Dunn post-hoc multiple comparison test (R package ‘Dunn’) 5 min. ((a) below diagonal), 45 min. ((a) above diagonal), 90 min. ((b) below diagonal), and 180 min. ((b) above diagonal) after electric-shock treatment.

(a)

| 5 min. and 45 min. | Control | 1 | 3 | 6 |
| --- | --- | --- | --- | --- |
| Control |  | *-0.39 (0.24)* | ***-3.63 (0.001)*** | ***-4.67 (<0.0001)*** |
| 1 | -1.21 (0.33) |  | *-2.27 (0.04)* | ***-3.32 (0.002)*** |
| 3 | **-3.56 (0.0014)** | -2.37 (0.03) |  | *-1.05 (0.36)* |
| 6 | **-4.54 (<0.0001)** | **-3.36 (0.002)** | -0.99 (0.39) |  |

(b)

| 90 min. and 180 min. | Control | 1 | 3 | 6 |
| --- | --- | --- | --- | --- |
| Control |  | *-1.65 (0.24)* | *-2.51 (0.088)* | *-1.74 (0.30)* |
| 1 | -0.07 (1) |  | *-0.88 (0.70)* | *-0.09 (1)* |
| 3 | -1.38 (0.41) | -1.32 (0.34) |  | *0.79 (0.63)* |
| 6 | -2.28 (0.17) | -2.23 (0.09) | -0.91 (0.53) |  |


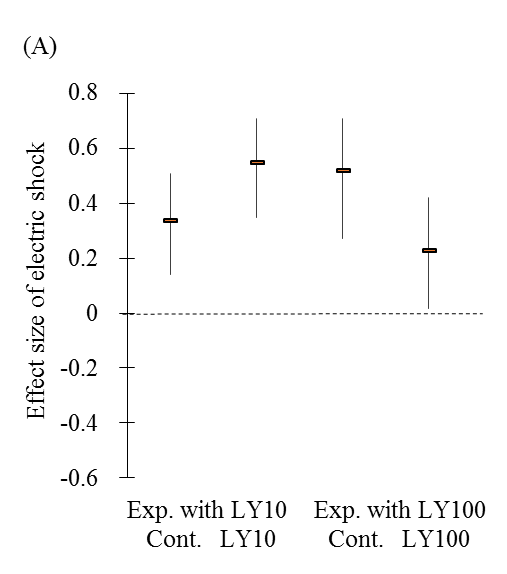


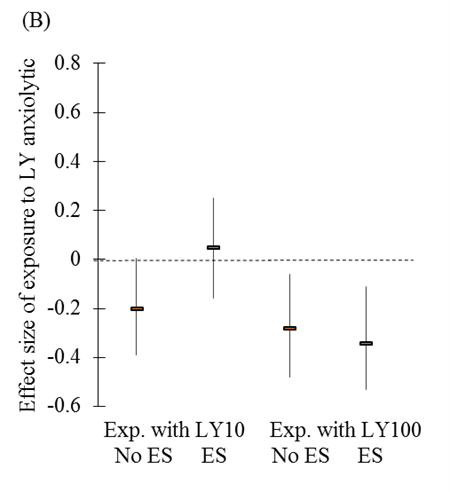


(C)


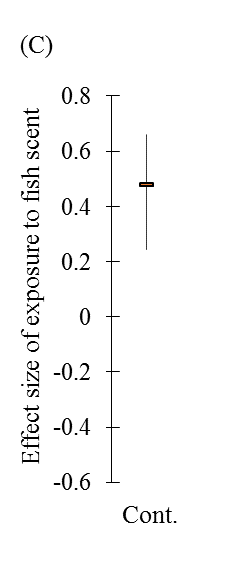


**Fig. S1:** **Effect of chronic exposure to LY anxiolytic on the use of refuge and the behavioral response to electric shock.** Effect size is estimated using the Cliff’s Delta index (median and bootstrapped IC (95%); package ‘Orddom’), and is considered as non-null if the IC does not cross zero. Positives values indicate an increase in refuge use, while negatives ones indicate the reverse. LY354740 was used at two concentrations, 10 µg.L^-1^ (LY10) and 100 µg.L^-1^ (LY100).

1. Effect size of exposure to electric shock in gammarids previously exposed (LY) or unexposed (Cont.) to the anxiolytic LY354740,
2. Effect size of exposure to the anxiolytic without (No ES) or with (ES) subsequent electric shock.
3. Effect size of exposure to the scent of a predatory fish (the brown trout *Salmo truttae*) on the use of refuge
