# Supplementary material for: Anxiety-like behaviour increases safety from fish predation in an amphipod crustacea
Source: R Soc Open Sci. 2017 Dec 6;4(12):171558. doi: 10.1098/rsos.171558 (PMC5750038; doi:10.1098/rsos.171558)
Supplement: Effect of electric-shock and anxiolytic treatments on locomotor activity [file rsos171558supp2.docx]

*Royal Society Open Science*

Supporting Information

Anxiety-like behaviour increases safety from fish predation in an amphipod crustacea

Marie-Jeanne Perrot-Minnot*, Loan Banchetry, Frank Cezilly

**Appendix 2**: Effect of electric-shock and anxiolytic treatments on locomotor activity


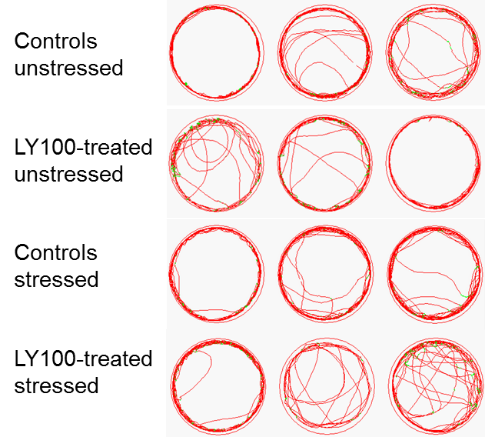


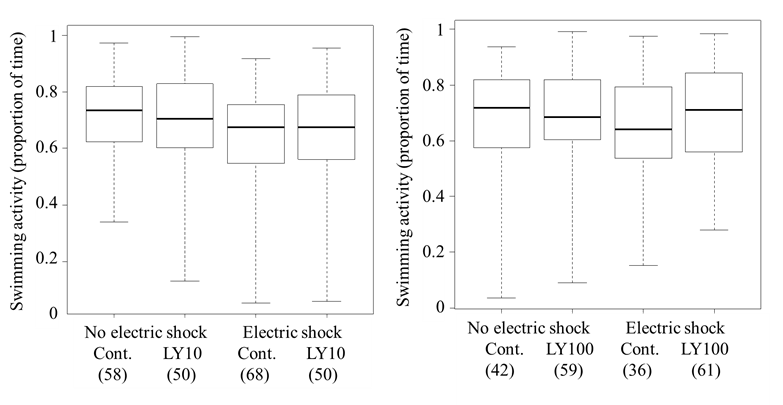


**Fig. S2** **Effect of electric shock and of anxiolytic treatment on swimming activity.** The influence of both treatments on the proportion of time spent swimming above a speed threshold of 15 mm.sec^-1^., was assessed using a two way ANOVA on arcsin-tranformed data.

1. Example cumulative traces of the swimming activity of gammarids exposed or not to electric shock and/or to anxiolytic treatment (3 individuals / treatment).
2. Swimming activity is not significantly affected by stress nor anxiolytic treatment, in the experiment with LY354740 at 10 µg.L^-1^ (LY10) and 100 µg.L^-1^ (LY100). Cont.: controls unexposed to LY.
